# Matrigel inhibits elongation and drives endoderm differentiation in aggregates of mouse embryonic stem cells

**DOI:** 10.1002/2211-5463.70044

**Published:** 2025-04-19

**Authors:** Atoosa Amel, Rachel Brown, Alexa Rabeling, Mubeen Goolam

**Affiliations:** ^1^ Department of Human Biology University of Cape Town South Africa; ^2^ UCT Neuroscience Institute Cape Town South Africa

**Keywords:** embryonic morphogenesis, embryonic stem cells, extracellular matrix, Matrigel, stembyro

## Abstract

Modelling peri‐implantation mammalian development using the self‐organising properties of stem cells is a rapidly growing field that has advanced our understanding of cell fate decisions occurring in the early embryo. Matrigel, a basement membrane matrix, is a critical substrate used in various protocols for its efficacy in promoting stem cell growth and self‐organisation. However, its role in driving stem cell lineage commitment, and whether this effect is driven by biochemical or physical cues, is not currently clear. Here, we grow embryoid bodies in suspension, Matrigel and agarose, an inert polysaccharide, to attempt to decouple the physical and biochemical roles of Matrigel and better understand how it drives stem cell differentiation. We use a combination of light microscopy, quantitative PCR and immunostaining to investigate gene and protein changes in our different culture conditions. We show that stem cell aggregates in Matrigel are hindered in their ability to elongate compared with those grown in agarose or in suspension, indicating that prohibitive role in self‐organisation. Aggregates in Matrigel are also driven to differentiate into endoderm, with ectoderm differentiation inhibited. Furthermore, these effects are not due to the physical presence of Matrigel, as the same effects are not witnessed in aggregates grown in agarose. Our results thus indicate that Matrigel has a significant and complex effect on the differentiation and morphology of embryoid bodies.

## Graphical abstract

Stem cell‐based embryo models (SCBEMs) are valuable to study early developmental milestones. Matrigel, a basement membrane matrix, is a critical substrate used in various SCBEM protocols, but its role in driving stem cell lineage commitment is not clearly defined. Here, we grow embryoid bodies in suspension, Matrigel and agarose to attempt to better understand how Matrigel drives stem cell differentiation. Created with BioRender.com.

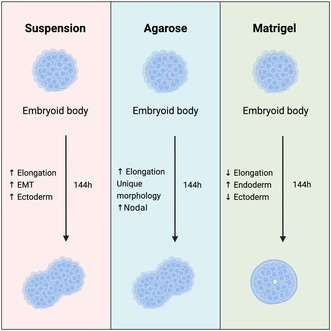

**Abbreviations**
A‐P: anterior–posterior
EBs: embryoid bodies
ECM: extracellular matrix
EMT: epithelial‐to‐mesenchymal transition
FBS: fetal bovine serum
FGF: fibroblast growth factor
iPS: induced pluripotent stem
LIF: leukaemia inhibitory factor
mES: mouse embryonic stem
mRNA: messenger ribonucleic acid
PBS: phosphate buffered saline
PCR: polymerase chain reaction
PFA: paraformaldehyde
PS: primitive streak
qPCR: quantitative polymerase chain reaction
SCBEMs: stem cell‐based embryo models

In mammals, the establishment of the body plan begins with gastrulation, a process initiated in the posterior region of the embryo through the appearance of the primitive streak (PS) that establishes the formation of the endoderm, mesoderm and ectoderm [1, 2, 3, 4]. Studying these early key developmental milestones is made challenging in mammals, as implantation into the uterine lining renders the embryo inaccessible for direct experimentation and manipulation. However, understanding these early self‐organising events remains a fundamental field of study in developmental biology.

Recent advances in stem cell culture techniques have revolutionised the study of early embryonic development as we are now able to study early embryonic milestones using *in vitro* stem cell‐based embryo models, so‐called ‘stem cell‐based embryo models (SCBEMs)’ [5, 6, 7, 8, 9, 10, 11, 12, 13]. A variety of ‘SCBEM’ techniques have been developed in a short space of time and have been shown to be able to resemble the blastocyst [14, 15, 16, 17], undergo gastrulation [18, 19], develop an anterior–posterior axis [20, 21, 22], develop a primitive neural tube [23, 24, 25] or even undergo the early stages of somitogenesis [23, 26] and even cardiogenesis [27, 28].

During the development of these protocols, one of the critical factors found to drive stem cell self‐organisation has been the addition of Matrigel. Matrigel is an extracellular matrix (ECM) derived from Engelbreth‐Holm‐Swarm mouse sarcoma [29, 30] that contains glycoproteins, proteoglycans, and growth factors and has been found to support embryo culture [31]. Used at different concentrations, the addition of Matrigel can drive ‘SCBEM’ elongation, somitogenesis and neural tube development in mouse embryonic stem (mES) cell‐based models [23, 26] as well as human induced pluripotent stem (iPS) cell‐based models referred to as somitoids [32] or axioloids [33]. These findings suggest that Matrigel plays a key role in driving stem cell differentiation in ‘SCBEMs’. However, Matrigel has a complex and unstandardised composition, and its composition can vary from batch to batch [34].

This variability may affect the reproducibility of experiments using Matrigel. Furthermore, Matrigel provides both structural support as well as a host of growth factors and signalling molecules making it unclear whether the advanced morphologies noted in ‘SCBEM’ cultures with Matrigel are due to the presence of mechanical or biochemical cues. Mechanical forces provided by a cell's environment have been shown to influence cell fate and properties. It has been demonstrated that a decrease in membrane tension brings about the exit from naïve pluripotency in mES cells by facilitating increased endocytosis of FGF signalling components [35]. Furthermore, ECM elasticity has been shown to influence cell fate commitment in naïve mesenchymal stem cells [36] as well as human embryonic stem cells [37]. These studies highlight the critical role of mechanical cues in regulating cellular behaviour. To uncouple these components, an inert material is needed to evaluate the influence of just mechanical cues on stem cell morphogenesis. Here, we investigate the role of mechanical constraints on morphological and gene expression changes in embryoid bodies (EBs), the simplest three‐dimensional aggregates of stem cells used to study their differentiation, when grown in suspension, Matrigel and agarose, an inert polysaccharide. In doing so we help to dissect the role of structural support on the fate of mES cells.

## Methods

### mES cell culture

In order to culture 129/Ola mES cells, a feeder layer of inactive murine embryonic fibroblast cells from Day 13 mouse embryos was cultured in 6‐well plates coated with 0.1% gelatin (G7041‐100G; Sigma‐Aldrich, Burlington, MA, USA) at a density of 1.6 × 10^4^ cells·cm^−2^ in a base medium containing DMEM (1×) + GlutaMAX™ (10566016; Gibco, Carlsbad, CA, USA), 15% FBS (10493106; Gibco), 0.05 mm β‐mercaptoethanol (21985023; Gibco) and 1% PenStrep (100 U penicillin/0.1 mg·mL^−1^ streptomycin, 15140122; Gibco). Feeder cells were allowed to settle in culture for 48 h before mES cells were seeded onto them. Two hours before seeding the mES cells, the medium was changed to 2i + LIF medium consisting of base medium supplemented with CHIR99021 (Chiron, 3 μm, SML1046‐5MG; Sigma‐Aldrich), PD0325901 (1 μm, PZ0162‐5MG; Sigma‐Aldrich) and Leukaemia inhibitory factor (10 ng·mL^−1^, LIF, A35934; Thermo Fisher Scientific, Waltham, MA, USA). The mES cells were then seeded onto the feeder layer at a density of 7 × 10^3^ cells·cm^−2^, and the medium was changed daily, with cells being passaged every other day. Cells were passaged using Dispase II (5 mg·mL^−1^, D4693‐1G; Sigma‐Aldrich, dissolved in DMEM (1×) + GlutaMAX™) that was added to the cells which were incubated for 20 min at 37 °C to facilitate lifting.

### Cell aggregates in suspension

The aggregate formation method was adopted from Bailie Johnson *et al*. [38]. mES cell colonies were treated with Dispase II (5 mg·mL^−1^, Sigma‐Aldrich D4693‐1G) at 37 °C for 20 min, followed by inactivation of Dispase and transfer of the cell suspension to a 15‐mL tube. The cells were centrifuged and resuspended in PBS to remove residual medium. Following the PBS washes, the cells were resuspended in N2B27 medium, which consists of base medium supplemented with 1% N2 supplement (17502048; Gibco) and 1% B27 plus supplement (A3582801; Gibco). The solution was diluted in N2B27 medium to give 1 × 10^4^ cells·mL^−1^. The suspension was then added in 40 μL droplets per well to a nonadhesive 96‐well U‐bottom plate (650185; Greiner Bio‐One, Kremsmünster, Austria). The plate was incubated at 37 °C and 5% CO_2_ for 48 h, after which a Chiron pulse (CHIR99021, 3 μm) was added to the aggregates and the plate was incubated for an additional 24 h. The following day, the Chiron pulse was removed and fresh N2B27 medium was added. The medium was changed every day until the required endpoint and not more than 168 h.

### Cell aggregates in agarose

The 96‐well flat bottom plates were coated with 30 μL of 1.2% agarose solution (1.2% w/v of Lonza Bioscience 50004 agarose added to deionised water and autoclaved) and left to dry for 10 min at RT. mES cell aggregates were made as above; however, instead of adding the cell suspension to 96‐well U‐bottom plates, 40 μL drops were placed in each well of the agarose‐containing plate. The plate was incubated at 37 °C and 5% CO_2_ for 48 h, after which a Chiron pulse (3 μm) was added to the aggregates, and they were incubated for an additional 24 h. The following day, the Chiron pulse was removed and fresh N2B27 medium was added. The medium was changed every day until the required endpoint and no longer than 168 h.

### Cell aggregates in Matrigel

mES cells were lifted using Dispase II after a 20 min incubation period, and the pellet was washed twice in PBS before counting. The cells were counted, and the appropriate volume was aliquoted and spun down again to give a concentration of 2 × 10^4^ cells·mL^−1^. The pellet was resuspended in 1 mL of Matrigel (356234; Corning, Corning, NY, USA). Droplets of 20 μL were evenly placed on a 10 cm culture plate, and Matrigel was left to solidify for 5 min at 37 °C and subsequently covered with N2B27 medium. The Chiron pulse was added to the medium in the dish on Day 2 of the culture and removed after 24 h. The medium was changed every second day until the required endpoint, but not more than 168 h.

### RNA extraction and cDNA synthesis

RNA was collected from aggregates grown in suspension, agarose or Matrigel at 144 h postaggregation using the High Pure RNA Isolation Kit (11828665001; Roche, Basel, Switzerland) following the manufacturer's instructions. Due to the limited amount of material obtained from each experiment, two experiments were combined to form one biological replicate. This was done for a total of two biological replicates per condition. The ImProm‐II™ Reverse Transcription System (Promega) was utilised for cDNA synthesis. A minimum of 1 μg of RNA was combined with 1 μL of Oligo (dT)15 primer (C110B; Promega) and nuclease‐free water to make up a total of 5 μL and incubated at 70 °C and 4 °C for 5 min each. PCR master mix made up of 6.1 μL nuclease‐free water, 4 μL 5× reaction buffer (M289A; Promega), 2.4 μL MgCl_2_ (A351H; Promega), 1 dNTP mix (C114B; Promega), 0.5 μL Recombinant RNasin® Ribonuclease Inhibitor (N251A; Promega), and 1 μL Reverse Transcriptase (M314A; Promega), was added to each sample. Thermal cycling was as follows: 25 °C for 5 min, 42 °C for 60 min and 70 °C for 15 min.

### qPCR

The StepOnePlus™ Real‐Time PCR System was used for quantitative PCR with SYBR green PCR Master‐Mix (4368708; Thermo‐Fisher Scientific). Primer sequences are available in Table 1. Briefly, 2 μL of cDNA (diluted 1 : 1 with nuclease‐free water) was mixed with 8 μL Master‐Mix, which consisted of: 5 μL SYBR green Master‐Mix, 0.4 μL of 10 μm forward and reverse primer mixture (5 μL of 100 μm primer stock with 40 μL nuclease‐free water), and 2.6 μL nuclease‐free water. Each reaction had three technical replicates. The run parameters can be found in Table 2. Gapdh was used as a housekeeping gene to calculate relative expression via the $2^{-\Delta \Delta C_{t}}$ method. Expression was normalised to cell aggregates cultured in base medium that did not receive a Chiron pulse. Data analysis was performed using MS Excel, while statistical analysis and graph generation were done using graphpad prism8 (Boston, MA, USA).

**Table 1:** Primers.

Gene	Forward sequence (5′ to 3′)	Reverse sequence (5′ to 3′)	Amplicon size (bp)
*Gapdh*	AGGTCGGTGTGAACGGATTTG	TGTAGACCATGTAGTTGAGGTCA	123
*Sox2*	GCGGAGTGGAAACTTTTGTCC	CGGGAAGCGTGTACTTATCCTT	157
*Sox17*	GATGCGGGATACGCCAGTG	CCACCACCTCGCCTTTCAC	136
*Gata6*	GTGGTCGCTTGTGTAGAAGGA	TTGCTCCGGTAACAGCAGTG	105
*Brachyury*	GCTGGATTACATGGTCCCAAG	GGCACTTCAGAAATCGGAGGG	158
*Mixl1*	GTCTTCCGACAGACCATGTACC	CCCGCCTTGAGGATAAGGG	160
*Pou3f1*	TTCAAGCAACGACGCATCAA	TGCGAGAACACGTTACCGTAGA	86
*Slc7a3*	TTCTGGCCGAGTTGTCTATGTTTG	AGTGCGGTTCTGTGGCTGTCTC	190
*E‐cadherin*	CAGGTCTCCTCATGGCTTTGC	CTTCCGAAAAGAAGGCTGTCC	175
*Nodal*	CCTGGAGCGCATTTGGATG	ACTTTTCTGCTCGACTGGACA	155
*Snai1*	CTTGTGTCTGCACGACCTGT	ACATCCGAGTGGGTTTGGAG	167
*Pax6*	TACCAGTGTCTACCAGCCAAT	TGCACGAGTATGAGGAGGTCT	194
*ß‐catenin*	ATGGAGCCGGACAGAAAAGC	CTTGCCACTCAGGGAAGGA	108
*Eomes*	TCGCTGTGACGGCCTACCAA	AGGGGAATCCGTGGGAGATGGA	210
*Wnt3*	CTCGCTGGCTACCCAATTTG	CTTGACACCTTCTGCTACGCT	165

**Table 2:** StepOnePlus™ Real‐Time PCR run parameters for a 2 h run.

Stage	Step	Temperature (°C)	Duration
Holding	1	95	10 min
Cycling (40 cycles)	1	95	15 s
	2	60	1 min
Melt Curve (continuous)	1	95	15 s
	2	60	1 min
	3	95 (with a ramp rate of 2.8%)	15 s

### Immunofluorescence

Cell aggregates collected at 144 h were fixed at 4 °C in 4% PFA for 1 h on a shaker. Subsequently, the aggregates were washed three times for 5–10 min with PBST (PBS, 0.05%; Tween‐20) on a rotating shaker and were permeabilised with 0.5% Triton‐X‐100 in PBS at room temperature for 1 h. After a brief washing step with PBST (three times for 5–10 min), the aggregates were blocked with PBS, 10% FBS and 0.2% Triton‐X‐100 at room temperature for 1 h. Primary antibody incubation was performed overnight at 4 °C in blocking buffer. The following day, the aggregates were washed three times quickly with PBS, followed by three 20 min washes. Secondary antibody incubation was performed overnight at 4 °C in blocking buffer, after which the washing procedure was repeated. Hoechst was added to the 20 min washes following secondary antibody incubation, and the samples were left to incubate overnight at 4 °C. Prior to imaging, aggregates were mounted onto Mowiol drops in glass‐bottomed dishes. Antibody details and dilutions used are provided in Table 3.

**Table 3:** Antibodies.

Antibody (source)	Supplier	Cat. #	Dilution
Hoechst	Thermo Fisher Scientific	H1399	1 : 1000
Anti‐human/mouse Brachyury (goat)	R&D Systems (Minneapolis, MN, USA)	AF2085	1 : 100
Anti‐mouse E‐cadherin (mouse)	Takara Bio Inc., (Kusatsu, Japan)	M108	1 : 500
Anti‐human SOX17 (goat)	R&D Systems	AF1924	1 : 100
Cy3 (donkey anti‐goat)	AEC‐Amersham SOC Ltd, (Midrand, South Africa)	705166147	1 : 500
Alexa 488 (donkey anti‐mouse)	AEC‐Amersham SOC Ltd	715546150	1 : 500

### Image analysis

The EVOS™ M5000 Imaging System microscope (Thermo Fisher Scientific) was used to capture images at a magnification of 10×. imagej (Madison, WI, USA) was used to optimise the brightness and contrast of all images and for measurements. The morphological characterisation was based on live images taken from a set number of wells every 24 h for the duration of 144 h. The outlines of the entire aggregate were traced manually to determine the perimeter, while the minor and major axial lengths were determined using the line tool. The elongation index was calculated by dividing the major axial length by the minor axial length. Multichannel images were taken using a Zeiss LSM 880 Airyscan confocal microscope (Zeiss, Oberkochen, Germany) using a 20× or a 40× water‐immersion objective. Z‐stack slices were spaced at 0.4 μm and the final image was deconvolved and displayed as a maximum projection using the open‐source ImageJ2 platform.

### Rheology

A total of100 μL droplets of Matrigel and various agarose concentrations (*n* = 5) were plated onto a preheated stage of a Kinexus Pro rheometer (Malvern Instruments, Malvern, UK) and allowed to form a gel. Frequency sweeps were performed at 37 °C, from 0.5 to 5 Hz, and the elastic component of the storage modulus (G′) was measured. All measurements were compared at 1 Hz.

### Statistical analysis

Statistical analyses were carried out using the functions provided by graphpad prism 8.4.2 (679). Because of the scarcity of biological material, two biological replicates (each consisting of two pooled experiments) were used for statistical analysis. When appropriate, an unpaired, nonparametric Mann–Whitney test was performed. Alternatively, a two‐way ANOVA was performed followed by a Fisher's least significant difference test. In all cases, the error bars represent the mean ± standard error of the mean (mean ± SEM). *P* values are represented as follows: ns = *P* ≤ 0.1234, * = *P* ≤ 0.0332, ** = *P* ≤ 0.0021, *** = *P* ≤ 0.0002, **** = *P* < 0.0001.

## Results

### The effect of Matrigel and agarose on the morphology of mES cell aggregates

**Fig. 1:**
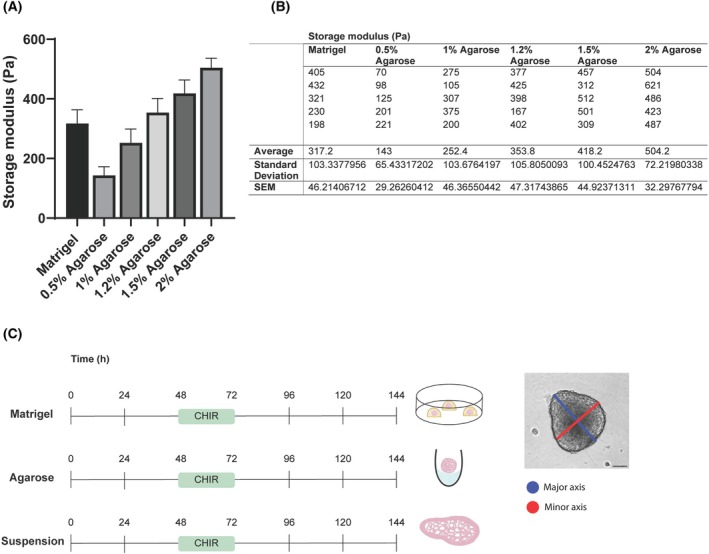
Storage modulus values for Matrigel and various agarose gels and experimental plan. (A) 1.2% agarose gel was found to have the most similar storage modulus to that of Matrigel, and this was used in further experiments. (B) Storage modulus (Pa), average storage modulus as well as standard deviation and SEM for all conditions. Error bars indicate mean + SEM, *n* = 5. (C) Embryonic stem (ES) cell aggregates were cultured in suspension, on agarose or embedded in Matrigel. All aggregates received a 24 h Chiron pulse on the third day of culture. To calculate the elongation of aggregates, the major axis (the longest axis; blue line) was measured and divided by the minor axis—the line perpendicular to the major axis at its midpoint (red line).

**Fig. 2:**
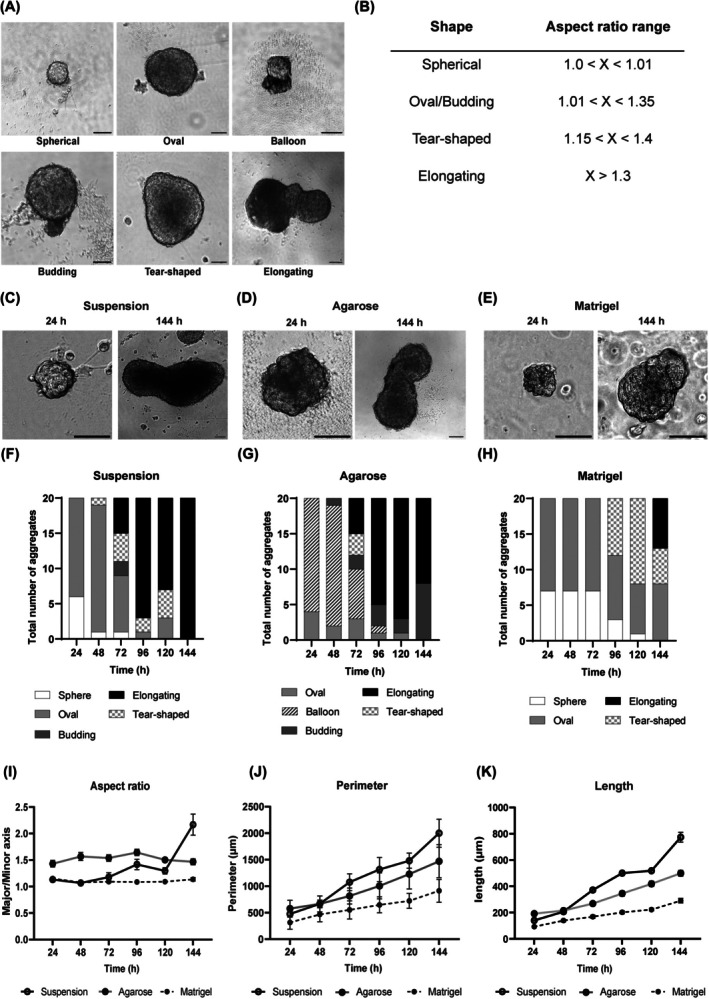
Comparative analysis of the effect of various physical constraints on embryonic stem (ES) cell aggregate morphology. (A) Spherical, oval, balloon, budding and tear‐shaped morphologies observed in aggregates. The balloon morphology was unique to aggregates cultured on agarose. (B) Aspect ratio used to classify aggregates. Aggregates were categorised as spherical, ovoid, tear‐shaped, budding or elongating based on their appearance and aspect ratio. (C) Morphology of aggregates in suspension. Aggregates had elongated by 144 h. (D) Morphology of aggregates cultured on agarose. At 24 h, the aggregates had already shown signs of asymmetry, and by 144 h, the aggregates had elongated. (E) Morphology of aggregates embedded in Matrigel. The aggregates were relatively smaller than the other two conditions and did not elongate significantly. (F) Characteristics of *n* = 20 aggregates grown in suspension for the period of 144 h. Major morphological changes occurred at 72 h, and at 144 h, all the aggregates had elongated. (G) Characteristics of *n* = 20 aggregates grown on agarose for the period of 144 h. The aggregates had a unique balloon‐like morphology in the first 96 h of culture, and by 144 h, some aggregates were still in the budding stage and had not elongated fully. (H) Characteristics of *n* = 20 aggregates grown in Matrigel for the period of 144 h. Aggregates had a delayed growth and only showed signs of asymmetry at 96 h. (I) Aspect ratio of aggregates in suspension (black line, empty circle), on agarose (grey line, dotted circle) and in Matrigel (dotted line, solid circle) for *n* = 20 aggregates per condition. (J) Perimeter of aggregates in suspension (black line, empty circle), on agarose (grey line, dotted circle) and in Matrigel (dotted line, solid circle) for *n* = 20 aggregates per condition. (K) Length of aggregates (μm) in suspension (black line, empty circle), on agarose (grey line, dotted circle) and in Matrigel (dotted line, solid circle) for *n* = 20 aggregates per condition. Error bars indicate mean ± SEM. Scale bar: 100 μm.

To examine the role of physical support in mES cell differentiation, we grew aggregates of ES cells in suspension, on agarose, and in Matrigel. Rheology was used to find the most appropriate agarose concentration to mimic the stiffness of Matrigel. We found that a 1.2% agarose gel had the most similar storage modulus to that of Matrigel and so was used in all further experiments (Fig. 1A,B). Under all conditions, EBs were subjected to a pulse of Chiron at 48 h for 24 h, and shape changes were quantified over time by measuring the aspect ratio of the aggregates (the longest axis of the aggregate, the major axis, divided by the distance perpendicular to the midpoint of the longest axis, the minor axis) (Fig. 1C). Aggregates were also classified based on visual characteristics and categorised as spherical, ovoid, tear‐shaped, budding or elongating based on their appearance and aspect ratio (Fig. 2A,B). While aggregates grown in suspension (Fig. 2C,F) or agarose (Fig. 2D,G) started to elongate at 72 h, following a pulse of Chiron, those grown in Matrigel primarily retained their spherical or ovoid structures at the same time (Fig. 2E,H). By 144 h, all aggregates in suspension (Fig. 2C,F) and 8/20 of those in agarose (Fig. 2D,G) were able to elongate. However, aggregates in Matrigel retained a more oval shape by the end of the culture period and did not elongate as easily (Fig. 2E,H). It was also noted that agarose aggregates had a unique, temporary balloon‐like morphology (Fig. 1A) that appeared at 24 h (16/20) and were mostly no longer present by 96 h (1/20) (Fig. 2G).

Aspect ratio analyses revealed that suspension aggregates were the only group that underwent a rapid period of elongation at 72 h and an additional increase in axial length at 144 h (Fig. 2I). Agarose aggregates were also found to have a higher starting aspect ratio from 24 h, likely due to these aggregates being initially deformed when placed in an agarose gel. Interestingly, there was a dip in aspect ratio at 120 h under both suspension and agarose conditions, meaning that the aggregates grew in width rather than length. The suspension aggregates had the highest rate of growth in perimeter, followed by the agarose aggregates, and the Matrigel embedded aggregates showed the smallest increase in perimeter (Fig. 2J) and length (Fig. 2K) during the 144 h period.

### Matrigel drives endoderm differentiation in mES cell aggregates

Physical support from the maternal environment plays an essential role in early embryonic development during the induction of the PS, when cells first undergo an epithelial‐to‐mesenchymal transition (EMT) and form mesodermal and endodermal progenitors [39]. In this study, we sought to determine the effects of physical constraints on the development of the PS and in driving EMT in EBs. To investigate this, we measured the expression of EMT and PS markers at 144 h using qPCR in EBs grown in suspension, agarose and Matrigel with a Chiron pulse, and compared this to EBs grown in suspension without any exposure to signalling molecules as a control sample.

**Fig. 3:**
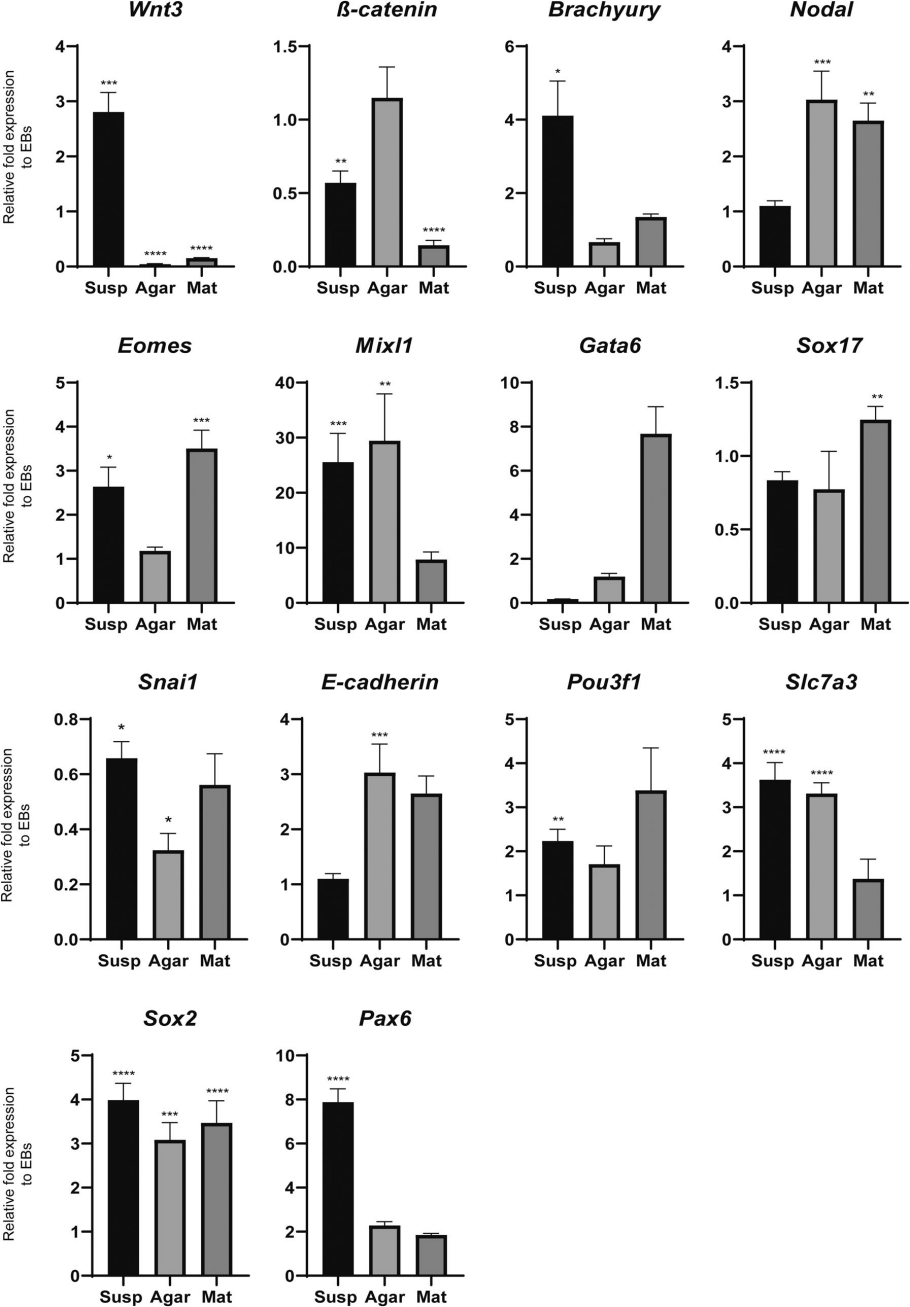
Effects of various physical constraints on gene expression of aggregates. qPCR was performed on reverse transcribed RNA extracted at 144 h on *n* = 100 aggregates over two biological repeats. Primitive streak markers: Wnt3, ß‐catenin, Brachyury and Nodal. Mesendoderm markers: Eomes and Mixl1. Endoderm markers: Gata6 and Sox17. EMT markers: Snai1 and E‐cadherin. Anterior/neurectoderm markers: Pou3f1, Slc7a3, Sox2 and Pax6. Relative fold expression to cell aggregates without a Chiron pulse. Error bars indicate mean ± SEM. An unpaired, nonparametric Mann–Whitney test was performed and *P ≤ 0.0332, **P ≤ 0.0021, ***P ≤ 0.0002, ****P < 0.0001.

The posterior epiblast marker Wnt3 was significantly increased in suspension aggregates compared with agarose and Matrigel aggregates, while there was little variation in the expression of ß‐catenin (Fig. 3). Brachyury, a marker of the PS and mesoderm, was also upregulated at 144 h under suspension conditions, with no increase in expression noted in agarose or Matrigel conditions (Fig. 3). However, another PS and mesoderm marker, Nodal, was significantly up‐regulated in both agarose and Matrigel conditions (Fig. 3). The mesendoderm marker Eomes was up‐regulated in EBs in suspension, as well as in Matrigel (Fig. 3), while another mesendoderm marker Mixl1 was significantly up‐regulated in suspension and agarose conditions but not in Matrigel (Fig. 3).

Interestingly, the early endoderm marker, Gata6, had increased levels of expression in Matrigel aggregates compared with the control (although not significantly), while a marker of the definitive endoderm (DE) marker, Sox17, was significantly increased in Matrigel compared with the other conditions, suggesting that Matrigel may be directing differentiation towards the endoderm lineage. Snai1, a key EMT marker, showed the highest expression under the suspension condition while exhibiting the lowest expression of the epithelial marker E‐cadherin. In contrast, the agarose condition displayed the lowest Snai1 expression and the highest E‐cadherin expression compared with other conditions (Fig. 3). The anterior epiblast marker Pou3f1 had higher levels of expression in all three conditions compared with controls, albeit with a significant increase only observed in suspension (Fig. 3). Similarly, Slc7a3, another anterior epiblast marker, had increased expression levels in all three conditions compared with controls, with a significant increase in suspension and agarose conditions (Fig. 3). The anterior/neuroectoderm marker Sox2 had an increased relative level of expression in the three conditions, while Pax6, a late neurectoderm marker, was only up‐regulated in suspension aggregates. This suggests that neuroectoderm formation may be at a more advanced stage in suspension aggregates exposed to a Chiron pulse.

### Aggregates in Matrigel express high levels of Brachyury protein

**Fig. 4:**
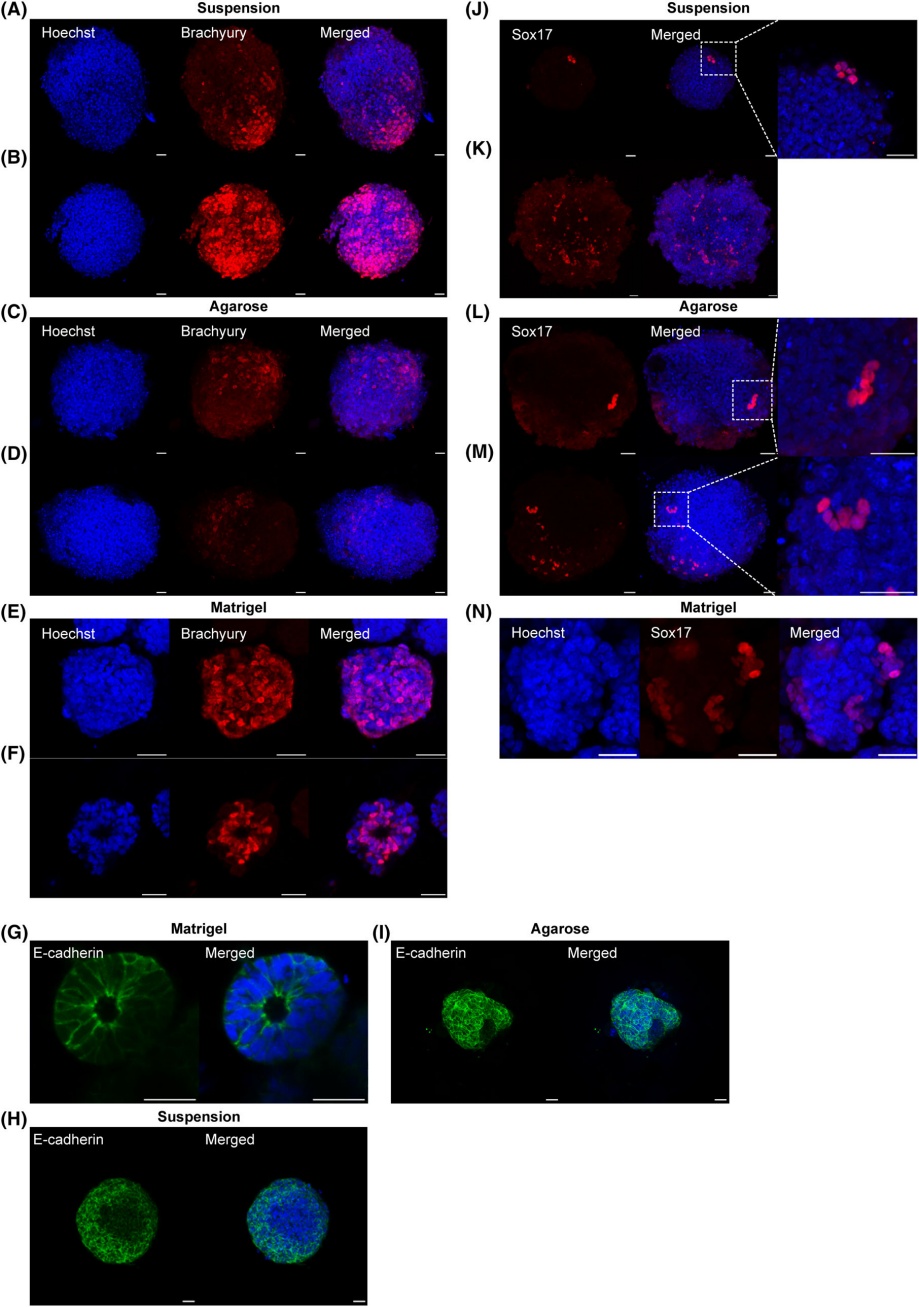
Immunofluorescence staining of Brachyury, Sox17 and E‐cadherin in aggregates cultured under various physical constraints. (A) Localised Brachyury positive cells at the elongating tip of suspension aggregates. (B) Brachyury positive cells spread throughout suspension aggregates that remained spherical (C) Localised Brachyury positive cells in agarose aggregates. (D) Dispersed Brachyury‐positive cells in agarose aggregates. (E) Brachyury positive cells dispersed throughout Matrigel aggregates. (F) Brachyury positive cells lining the lumen of Matrigel aggregates. (G) Epithelial‐like cells surrounding the cavity in Matrigel aggregates stained positive for epithelial marker E‐cadherin. (H) Suspension aggregates stained for E‐cadherin had regions with reduced expression that may represent EMT. (I) Agarose aggregates stained for E‐cadherin had regions with reduced expression that may represent EMT. (J) Pockets of Sox17 positive cells in the periphery of suspension aggregates. (K) Sox17 positive cells dispersed across the surface of suspension aggregates. (L) Pockets of Sox17 positive cells in aggregates cultured on agarose. (M) Sox17 positive cells dispersed across the surface of aggregates grown on agarose. (N) Sox17 positive cells in dispersed groups across the surface of aggregates in Matrigel. Scale bar: 25 μm. Suspension aggregates *n* = 50, agarose aggregates *n* = 46, Matrigel aggregates *n* = 60.

The effects that physical constraints had on mesoderm and endoderm gene expression next led us to investigate the spatial expression of the mesoderm marker Brachyury and the endoderm marker Sox17 in our aggregates using immunofluorescence. When in suspension very few aggregates expressed Brachyury. When they did, this expression was either restricted to one pole of the aggregate (Fig. 4A, 1/50) or dispersed throughout the entire aggregate (Fig. 4B, 2/50). A highly similar pattern was observed in the agarose aggregates with only a few aggregates being positive for Brachyury, and when they were, this staining was either localised (Fig. 4C, 2/46) or dispersed (Fig. 4D, 2/46).

Aggregates in Matrigel, however, have significantly more Brachyury positive aggregates, with 18/60 showing a dispersed pattern of expression (Fig. 4E). Interestingly, all of these also developed a Brachyury lined lumen in their centres (Fig. 4F, 18/60). All aggregates in Matrigel were found to have formed a similar cavity, even those without any Brachyury‐positive staining. The cells surrounding these cavities appeared epithelial‐like and were brightly stained with E‐cadherin (Fig. 4G). The suspension and agarose aggregates clearly lacked such cavities, but they had regions that were negative for E‐cadherin (Fig. 4H,I) suggesting that EMT has occurred.

Endoderm staining was observed infrequently in all conditions investigated. In suspension aggregates, it was noted as either small pockets of Sox17‐positive cells near the periphery (Fig. 4J, 7/46) or dispersed across the surface of the aggregate (Fig. 4K, 7/46). A similar expression pattern was observed in agarose aggregates with small pockets of Sox17‐positive cells (Fig. 4L, 3/49) or a dispersed expression pattern (Fig. 4M, 5/49). Aggregates in Matrigel were noted to express Sox17 in dispersed groups across the surface of the aggregate (Fig. 4N, 5/60).

## Discussion

The emergence and subsequent development of ‘SCBEM’ culture systems has generated a noninvasive, scalable and novel way to investigate early mammalian embryonic morphogenesis and cell fate decisions, gastrulation, and germ layer formation in mammals. One of the key aspects missing from ‘SCBEM’ structures is the presence of physical support provided by the ECM normally generated by the basement membrane of the visceral endoderm. To address this issue, many of these systems incorporate the commercial basement membrane substitute Matrigel. Although Matrigel can promote stem cell self‐organisation and provides some degree of mechanical constraint, it is ill‐defined and suffers from lot‐to‐lot variability. In addition, its effect on organoid structure and differentiation is influenced by both mechanical and biochemical cues, and the specific component/s responsible for the observed morphologies remain unknown. Matrigel also does not support any controlled modifications of its stiffness or components, and thus, there is a need to use an inert and manipulatable scaffold to separate the effects of physical and chemical cues on stem cell self‐organisation in this burgeoning field. In this study, we used agarose, an inert polysaccharide, to test the role of mechanical cues on the fate of EBs and to try to create similar physical constraints to those provided by the uterus.

We found that EB morphology was significantly influenced by the physical constraints present during culture, with suspended aggregates exhibiting more effective growth and elongation than counterparts grown in the presence of Matrigel or agarose. At 48 h, some suspension and agarose aggregates began to exhibit tear‐shaped or budding structures, indicating an intrinsic breaking of symmetry. At 72 h, there was a sudden increase in the variety of morphologies, including balloon‐like structures, which were observed as intermediates in the elongation process. Interestingly, we found that the presence of Matrigel inhibited elongation, causing aggregates to maintain their oval or spherical shape for longer. When we measured aspect ratios, we found that aggregates in suspension underwent a drop in aspect ratio at 120 h, followed by a sudden increase at 144 h. This suggests that cell proliferation occurs before elongation along the major axis. Aggregates grown in agarose exhibited a similar dip and rise in aspect ratio, but the same pattern was not found in aggregates grown on Matrigel. Overall, we found that early self‐patterning events in stem cell aggregates in culture are hindered by the presence of Matrigel. These aggregates neither grow nor elongate as effectively as those in suspension or in agarose, with Matrigel playing a prohibitive role in self‐organisation.

Previous studies have shown that by 144 h in culture ‘SCBEM’ have undergone a gastrulation‐like process [20, 22, 23, 40]. Although we noted elongation in EBs in both suspension and agarose conditions by this time point, there were marked differences in the expression of key markers between the two conditions. Aggregates in suspension had increased levels of *Brachyury* suggesting that the PS had been initiated. In contrast, aggregates grown in agarose did not have this same up‐regulation in *Brachyury* expression. They did, however, have up‐regulated *ß‐catenin*, an upstream regulator of *Brachyury*. This could indicate a delay in the gastrulation process due to the differences in the physical properties of the environments as the more restrictive agarose may present a less conducive environment for elongation and subsequent gastrulation. Aggregates in Matrigel had similarly low levels of *Brachyury* and *ß‐catenin*, further supporting the theory that physical support hinders stem cell driven *in vitro* gastrulation‐like events. Aggregates in suspension also had the lowest relative levels of *E‐cadherin*, an epithelial marker, and the highest levels of mesenchymal marker *Snai1*, suggesting that these aggregates are likely undergoing EMT, a critical process in gastrulation. This is further support for suspension aggregates being more developmentally advanced than aggregates in either agarose or Matrigel.

However, the expression patterns of *Nodal* and *Wnt3* in our aggregates indicate that the developmental landscape in our conditions is more complex than this. *Nodal* and *Wnt3* are known to work together to induce the expression of *Brachyury* and *Eomes*, another marker of gastrulation [1]. We observed that *Nodal* and *Wnt3* have opposing expression patterns in suspension aggregates, with elevated levels of *Wnt3* and low levels of *Nodal*. Interestingly, the opposite pattern, high levels of *Nodal* and relatively low levels of *Wnt3*, was observed in aggregates in agarose and Matrigel. These results would indicate that the aggregates under these conditions are more advanced in terms of mesoderm differentiation. As *Eomes* has been shown to precede *Brachyury* expression [41], and based on the high levels of *Eomes*, we can assume that *Brachyury* expression would increase in both suspension and Matrigel aggregates if cultured further. As both aggregates in Matrigel and agarose show a similar pattern of expression in this case, we hypothesise that it is principally mechanical cues driving this expression pattern, and this warrants further investigation.

Analysing the qPCR expression of key markers of PS formation and EMT transition, it appears that our aggregates might most closely resemble mouse embryos at E5.5‐E6.5, between the emergence of the germ layers and the formation of the anterior–posterior axis (A‐P axis), which occurs at approximately 120–144 h of development [6]. It is noted that this assumption is based on our qPCR data and not a full transcriptomic analysis, which would be needed to confirm this developmental timing. With this in mind, we still find that aggregates are significantly delayed compared with other ‘SCBEM’ culture processes, where aggregates can undergo a gastrulation‐like process after 96 h and exhibit similarities to the E8.5 mouse embryo [20, 22, 23, 40].

Despite this apparent developmental delay, suspension aggregates had high levels of anterior/neuroectoderm markers, *Pou3f1, Slc7a3* and *Sox2*, indicating the initiation of the anterior–posterior (A‐P) axis. We observed a distinct increase in *Pax6*, a late neuroectoderm marker, in suspension aggregates similar to what is observed in E8.5 mouse embryos and gastruloids (one of the most robust ‘SCBEM’ systems) at 120 h [22]. This is possibly due to the high levels of *Sox2* present in mES cells prior to differentiation, allowing rapid neural differentiation [42], provided they were able to differentiate without any confounding effects, such as physical constraints.

Aggregates in Matrigel demonstrated the highest expression of endoderm markers compared with the other two conditions. *Gata6* is typically a marker for cardiac mesoderm and DE progenitors [43], while *Sox17* is a well‐known DE marker [44]. *Gata6* mRNA was up‐regulated in Matrigel aggregates, followed by up‐regulation of *Sox17*, indicating that this condition likely favoured endoderm formation. Upregulated *Gata6* mRNA expression could also be an indicator of cardiac crescent cells, which have also been observed in the anterior side of gastruloids [22]. It is unclear whether this preference is due to physical restrictions imposed by Matrigel or the presence of certain signalling factors. However, evidence suggests that laminin, a major component of Matrigel, can direct human ES cells towards the endoderm lineage [45]. Furthermore, studies have demonstrated that culturing epiblast stem cells in Matrigel results in up‐regulated levels of endoderm‐related transcription factors, such as Sox17 [46], further supporting the notion that Matrigel influences lineage commitment towards endoderm.

Immunofluorescent staining for Brachyury revealed that the positive cells were located exclusively at the elongated pole of the aggregates in both suspension and agarose, as observed in other studies [20, 22, 40, 47], although the frequency of this pattern was significantly lower than reported by others. Aggregates in Matrigel, however, had many more aggregates that were Brachyury‐positive, and these cells were principally in the centre of the aggregate surrounding the lumen. This is in contrast to our mRNA expression results which indicated that Brachyury was lower in Matrigel aggregates than the other conditions. We hypothesise that this is due to mRNA being rapidly degraded once translated while protein is more stable. Studies have demonstrated that Matrigel can increase the expression of Wnt3 antagonists, such as Dkk1 and Sfpr1 [48]. Therefore, it is plausible that cells located in the periphery of our Matrigel aggregates express Wnt3 agonists, resulting in the localisation of Wnt3 activity in the centre of the aggregates, which leads to the up‐regulation of Brachyury expression. By embedding aggregates in Matrigel, we were able to observe cavity formation. This was likely due to the cells pulling apart, facilitated by E‐cadherin localisation around the membrane. Matrigel is used as a substitute for ECM derived from extra‐embryonic tissue [18] and the interaction between cells and Matrigel via integrin receptors facilitates cell polarisation and cavity formation [49].

We do also make note that although all conditions started with the same cell number, by 144 h our Matrigel aggregates were indeed smaller than the aggregates in suspension or agarose, which were of comparable size. We thus cannot rule out that these size differences, caused by culturing the aggregates in Matrigel, did not have a role to play in the differences in gene expression and cavitation we saw.

## Conclusions

Overall, our results suggest that Matrigel has a significant and complex effect on the differentiation and morphology of mES cells in culture. It can hinder stem cell self‐organisation and neural differentiation, and drive endoderm differentiation. Its effects are not driven simply by the mechanical force it provides as its effects on stem cells are not mimicked by using agarose. Although Matrigel is a critical component of ‘SCBEM’ cultures, caution should be taken when viewing it as a substitute for an ECM. Future research in the field should focus on finding a more defined and manipulatable replacement for Matrigel in order to advance the field and the accuracy of *in vitro* embryo models.

## Conflict of interest

The authors declare no conflict of interest.

## Peer review

The peer review history for this article is available at https://www.webofscience.com/api/gateway/wos/peer‐review/10.1002/2211‐5463.70044.

## Author contributions

AA, RB and AR contributed to the investigation. AA and MG contributed to the writing. MG contributed to the conceptualisation, funding acquisition, project administration and supervision.

## Data Availability

All data are provided in the manuscript. Any further enquiries can be directed to the corresponding author.

## References

[1] Arnold SJ and Robertson EJ (2009) Making a commitment: cell lineage allocation and axis patterning in the early mouse embryo. Nat Rev Mol Cell Biol 10, 91–103. 19129791 10.1038/nrm2618

[2] Stower MJ and Bertocchini F (2017) The evolution of amniote gastrulation: the blastopore‐primitive streak transition. Wiley Interdiscip Rev Dev Biol 6, e262. 10.1002/wdev.262 28177589

[3] Sheng G , Martinez Arias A and Sutherland A (2021) The primitive streak and cellular principles of building an amniote body through gastrulation. Science 374, abg1727. 34855481 10.1126/science.abg1727

[4] Tam PPL and Behringer RR (1997) Mouse gastrulation: the formation of a mammalian body plan. Mech Dev 68, 3–25. 9431800 10.1016/s0925-4773(97)00123-8

[5] Amel A , Rossouw S and Goolam M (2023) Gastruloids: a novel system for disease modelling and drug testing. Stem Cell Rev Rep 19, 104–113. 36308705 10.1007/s12015-022-10462-5

[6] Arias AM , Marikawa Y and Moris N (2022) Gastruloids: pluripotent stem cell models of mammalian gastrulation and embryo engineering. Dev Biol 488, 35–46. 35537519 10.1016/j.ydbio.2022.05.002 PMC9477185

[7] Veenvliet JV , Lenne PF , Turner DA , Nachman I and Trivedi V (2021) Sculpting with stem cells: how models of embryo development take shape. Development 148, dev192914. 34908102 10.1242/dev.192914 PMC8722391

[8] van den Brink SC and van Oudenaarden A (2021) 3D gastruloids: a novel frontier in stem cell‐based in vitro modeling of mammalian gastrulation. Trends Cell Biol 31, 747–759. 34304959 10.1016/j.tcb.2021.06.007

[9] Zylicz JJ (2020) Defined stem cell culture conditions to model mouse blastocyst development. Curr Protoc Stem Cell Biol 52, e105. 31971672 10.1002/cpsc.105

[10] Taniguchi K , Heemskerk I and Gumucio DL (2019) Opening the black box: stem cell‐based modeling of human post‐implantation development. J Cell Biol 218, 410–421. 30552099 10.1083/jcb.201810084 PMC6363460

[11] Shahbazi MN and Zernicka‐Goetz M (2018) Deconstructing and reconstructing the mouse and human early embryo. Nat Cell Biol 20, 878–887. 30038253 10.1038/s41556-018-0144-x

[12] Simunovic M and Brivanlou AH (2017) Embryoids, organoids and gastruloids: new approaches to understanding embryogenesis. Development 144, 976–985. 28292844 10.1242/dev.143529 PMC5358114

[13] Turner DA , Baillie‐Johnson P and Martinez Arias A (2016) Organoids and the genetically encoded self‐assembly of embryonic stem cells. Bioessays 38, 181–191. 26666846 10.1002/bies.201500111 PMC4737349

[14] Rivron NC , Frias‐Aldeguer J , Vrij EJ , Boisset J‐C , Korving J , Vivié J , Truckenmüller RK , van Oudenaarden A , van Blitterswijk CA and Geijsen N (2018) Blastocyst‐like structures generated solely from stem cells. Nature 557, 106–111. 29720634 10.1038/s41586-018-0051-0

[15] Sozen B , Cox AL , De Jonghe J , Bao M , Hollfelder F , Glover DM and Zernicka‐Goetz M (2019) Self‐Organization of Mouse Stem Cells into an extended potential Blastoid. Dev Cell 51, 698–712.e8. 31846649 10.1016/j.devcel.2019.11.014 PMC10291877

[16] Yu L , Wei Y , Duan J , Schmitz DA , Sakurai M , Wang L , Wang K , Zhao S , Hon GC and Wu J (2021) Blastocyst‐like structures generated from human pluripotent stem cells. Nature 591, 620–626. 33731924 10.1038/s41586-021-03356-y

[17] Heidari Khoei H , Javali A , Kagawa H , Sommer TM , Sestini G , David L , Slovakova J , Novatchkova M , Scholte op Reimer Y and Rivron N (2023) Generating human blastoids modeling blastocyst‐stage embryos and implantation. Nat Protoc 18, 1584–1620. 36792779 10.1038/s41596-023-00802-1 PMC7617227

[18] Harrison SE , Sozen B , Christodoulou N , Kyprianou C and Zernicka‐Goetz M (2017) Assembly of embryonic and extraembryonic stem cells to mimic embryogenesis in vitro. Science 356, eaal1810. 28254784 10.1126/science.aal1810

[19] Sozen B , Amadei G , Cox A , Wang R , Na E , Czukiewska S , Chappell L , Voet T , Michel G , Jing N *et al*. (2018) Self‐assembly of embryonic and two extra‐embryonic stem cell types into gastrulating embryo‐like structures. Nat Cell Biol 20, 979–989. 30038254 10.1038/s41556-018-0147-7

[20] van den Brink SC , Baillie‐Johnson P , Balayo T , Hadjantonakis AK , Nowotschin S , Turner DA and Martinez Arias A (2014) Symmetry breaking, germ layer specification and axial organisation in aggregates of mouse embryonic stem cells. Development 141, 4231–4242. 25371360 10.1242/dev.113001 PMC4302915

[21] Turner DA , Girgin M , Alonso‐Crisostomo L , Trivedi V , Baillie‐Johnson P , Glodowski CR *et al*. (2017) Anteroposterior polarity and elongation in the absence of extraembryonic tissues and spatially localised signalling in gastruloids, mammalian embryonic organoids. Development 144, 3894–3906. 28951435 10.1242/dev.150391 PMC5702072

[22] Beccari L , Moris N , Girgin M , Turner DA , Baillie‐Johnson P , Cossy A‐C , Lutolf MP , Duboule D and Arias AM (2018) Multi‐axial self‐organization properties of mouse embryonic stem cells into gastruloids. Nature 562, 272–276. 30283134 10.1038/s41586-018-0578-0

[23] Veenvliet JV , Bolondi A , Kretzmer H , Haut L , Scholze‐Wittler M , Schifferl D , Koch F , Guignard L , Kumar AS , Pustet M *et al*. (2020) Mouse embryonic stem cells self‐organize into trunk‐like structures with neural tube and somites. Science 370, eaba4937. 33303587 10.1126/science.aba4937

[24] Libby ARG , Joy DA , Elder NH , Bulger EA , Krakora MZ , Gaylord EA , Mendoza‐Camacho F , Butts JC and McDevitt TC (2021) Axial elongation of caudalized human organoids mimics aspects of neural tube development. Development 148, dev198275. 34142711 10.1242/dev.198275 PMC8254868

[25] Bérenger‐Currias NM , Mircea M , Adegeest E , van den Berg PR , Feliksik M , Hochane M , Idema T , Tans SJ and Semrau S (2022) A gastruloid model of the interaction between embryonic and extra‐embryonic cell types. J Tissue Eng 13, 20417314221103042. 35707767 10.1177/20417314221103042 PMC9189523

[26] van den Brink SC , Alemany A , van Batenburg V , Moris N , Blotenburg M , Vivié J , Baillie‐Johnson P , Nichols J , Sonnen KF , Martinez Arias A *et al*. (2020) Single‐cell and spatial transcriptomics reveal somitogenesis in gastruloids. Nature 582, 405–409. 32076263 10.1038/s41586-020-2024-3

[27] Rossi G , Broguiere N , Miyamoto M , Boni A , Guiet R , Girgin M , Kelly RG , Kwon C and Lutolf MP (2021) Capturing cardiogenesis in gastruloids. Cell Stem Cell 28, 230–240.e6. 33176168 10.1016/j.stem.2020.10.013 PMC7867643

[28] Olmsted ZT and Paluh JL (2022) A combined human gastruloid model of cardiogenesis and neurogenesis. iScience 25, 104486. 35721464 10.1016/j.isci.2022.104486 PMC9198845

[29] Kleinman HK and Martin GR (2005) Matrigel: basement membrane matrix with biological activity. Semin Cancer Biol 15, 378–386. 15975825 10.1016/j.semcancer.2005.05.004

[30] Kleinman HK , McGarvey ML , Hassell JR , Star VL , Cannon FB , Laurie GW and Martin GR (1986) Basement membrane complexes with biological activity. Biochemistry 25, 312–318. 2937447 10.1021/bi00350a005

[31] Carnegie J , Claman P , Lawrence C and Cabaca O (1995) Can Matrigel substitute for Vero cells in promoting the in‐vitro development of mouse embryos? Hum Reprod 10, 636–641. 7782445 10.1093/oxfordjournals.humrep.a136002

[32] Sanaki‐Matsumiya M , Matsuda M , Gritti N , Nakaki F , Sharpe J , Trivedi V and Ebisuya M (2022) Periodic formation of epithelial somites from human pluripotent stem cells. Nat Commun 13, 2325. 35484123 10.1038/s41467-022-29967-1 PMC9050736

[33] Yamanaka Y , Hamidi S , Yoshioka‐Kobayashi K , Munira S , Sunadome K , Zhang Y , Kurokawa Y , Ericsson R , Mieda A , Thompson JL *et al*. (2023) Reconstituting human somitogenesis in vitro. Nature 614, 509–520. 36543322 10.1038/s41586-022-05649-2

[34] Hughes CS , Postovit LM and Lajoie GA (2010) Matrigel: a complex protein mixture required for optimal growth of cell culture. Proteomics 10, 1886–1890. 20162561 10.1002/pmic.200900758

[35] De Belly H , Stubb A , Yanagida A , Labouesse C , Jones PH , Paluch EK *et al*. (2021) Membrane tension gates ERK‐mediated regulation of pluripotent cell fate. Cell Stem Cell 28, 273–284.e6. 33217323 10.1016/j.stem.2020.10.018 PMC7875115

[36] Engler AJ , Sen S , Sweeney HL and Discher DE (2006) Matrix elasticity directs stem cell lineage specification. Cell 126, 677–689. 16923388 10.1016/j.cell.2006.06.044

[37] Przybyla L , Lakins JN and Weaver VM (2016) Tissue mechanics orchestrate Wnt‐dependent human embryonic stem cell differentiation. Cell Stem Cell 19, 462–475. 27452175 10.1016/j.stem.2016.06.018 PMC5336327

[38] Baillie‐Johnson P , van den Brink SC , Balayo T , Turner DA and Martinez Arias A (2015) Generation of aggregates of mouse embryonic stem cells that show symmetry breaking, polarization and emergent collective behaviour in vitro. J Vis Exp 53252. doi: 10.3791/53252 26650833 PMC4692741

[39] Gattazzo F , Urciuolo A and Bonaldo P (2014) Extracellular matrix: a dynamic microenvironment for stem cell niche. Biochim Biophys Acta 1840, 2506–2519. 24418517 10.1016/j.bbagen.2014.01.010 PMC4081568

[40] Turner DA , Girgin M , Alonso‐Crisostomo L , Trivedi V , Baillie‐Johnson P , Glodowski CR , Hayward PC , Collignon J , Gustavsen C , Serup P *et al*. (2017) Anteroposterior polarity and elongation in the absence of extra‐embryonic tissues and of spatially localised signalling in gastruloids: mammalian embryonic organoids. Development 144, 3894–3906. 28951435 10.1242/dev.150391 PMC5702072

[41] Tosic J , Kim G‐J , Pavlovic M , Schröder CM , Mersiowsky S‐L , Barg M , Hofherr A , Probst S , Köttgen M , Hein L *et al*. (2019) Eomes and brachyury control pluripotency exit and germ‐layer segregation by changing the chromatin state. Nat Cell Biol 21, 1518–1531. 31792383 10.1038/s41556-019-0423-1

[42] Thomson M , Siyuan , Zou L‐N , Smith Z , Meissner A and Ramanathan S (2011) Pluripotency factors in embryonic stem cells regulate differentiation into germ layers. Cell 145, 875–889. 21663792 10.1016/j.cell.2011.05.017 PMC5603300

[43] Morrisey EE , Ip HS , Lu MM and Parmacek MS (1996) GATA‐6: a zinc finger transcription factor that is expressed in multiple cell lineages derived from lateral mesoderm. Dev Biol 177, 309–322. 8660897 10.1006/dbio.1996.0165

[44] Choi E , Kraus MRC , Lemaire LA , Yoshimoto M , Vemula S , Potter LA , Manduchi E , Stoeckert CJ Jr , Grapin‐Botton A and Magnuson MA (2012) Dual lineage‐specific expression of Sox17 during mouse embryogenesis. Stem Cells 30, 2297–2308. 22865702 10.1002/stem.1192 PMC3448801

[45] Wong JC , Gao SY , Lees JG , Best MB , Wang R and Tuch BE (2010) Definitive endoderm derived from human embryonic stem cells highly express the integrin receptors alphaV and beta5. Cell Adhes Migr 4, 39–45. 10.4161/cam.4.1.10627 PMC2852556 20026907

[46] Inamori S , Fujii M , Satake S , Iida H , Teramoto M , Sumi T , Meno C , Ishii Y and Kondoh H (2020) Modeling early stages of endoderm development in epiblast stem cell aggregates with supply of extracellular matrices. Develop Growth Differ 62, 243–259. 10.1111/dgd.12663 PMC7318635 32277710

[47] Girgin MU , Broguiere N , Mattolini L and Lutolf MP (2021) Gastruloids generated without exogenous Wnt activation develop anterior neural tissues. Stem Cell Reports 16, 1143–1155. 33891872 10.1016/j.stemcr.2021.03.017 PMC8185432

[48] Yamanaka Y , Hamidi S , Yoshioka‐Kobayashi K , Munira S , Sunadome K , Zhang Y , Kurokawa Y , Ericsson R , Mieda A , Thompson JL *et al*. (2022) Reconstituting human somitogenesis in vitro. Nature 614, 509–520. 36543322 10.1038/s41586-022-05649-2

[49] Bedzhov I , Graham SJ , Leung CY and Zernicka‐Goetz M (2014) Developmental plasticity, cell fate specification and morphogenesis in the early mouse embryo. Philos Trans R Soc Lond Ser B Biol Sci 369, 20130538. 25349447 10.1098/rstb.2013.0538 PMC4216461

